# Specific cognitive and psychological alterations are more strongly linked to increased migraine disability than chronic migraine diagnosis

**DOI:** 10.1186/s10194-024-01734-1

**Published:** 2024-03-15

**Authors:** Tatiana Castro Zamparella, Mariela Carpinella, Mario Peres, Florencia Patricia Cuello, Pilar Maza, Melanie Van Gansen, Marcelo Filipchuk, Verónica Balaszczuk, Carolina Maldonado, Pablo Scarnato, Diego Conci Magris, Marco Lisicki

**Affiliations:** 1 Neuroscience Unit, Conci-Carpinella Institute, 358 (5000) Córdoba, Urquiza Argentina; 2 Psychological Research Institute, Faculty of Psychology, National University of Córdoba (UNC - CONICET), Córdoba, Argentina; 3 National Council for Scientific and Technical Research (CONICET), Córdoba, Argentina; 4 Faculty of Psychology, National University of Córdoba (UNC), Córdoba, Argentina; 5 Faculty of Medicine, Catholic University of Cuyo, San Luis, Argentina; 6 Hospital Israelita Albert Einstein, São Paulo, SP Brazil; 7 Instituto de Psiquiatria, Hospital das Clínicas da Faculdade de Medicina da USP, Sao Paulo, SP Brazil; 8 Faculty of Exact, Physical and Natural Sciences, National University of Córdoba (UNC), Córdoba, Argentina; 9 Biomedical Physics Department, School of Medicine, National University of Córdoba (UNC), Córdoba, Argentina

**Keywords:** Chronic migraine, Disability, Burden, Voxel-based morphometry, Machine learning

## Abstract

**Introduction:**

The efficiency of The International Classification of Headache Disorders (ICHD-3) in reflecting patients’ disability has recently been questioned. This prompts consideration that clinical features beyond pain may more accurately indicate the extent of underlying brain impairment than the mere frequency of headache days. Important cognitive dysfunctions and psychological impairment have been reported in burdensome cases of migraine, and the presence of these alterations has been associated with biological changes in the nervous system. This study aimed to compare migraine-related disability within a specific patient group, classified using ICHD-3 criteria or classified based on findings from a neuropsychological evaluation using machine learning. Additionally, a complementary voxel-based morphometry (VBM) comparison was conducted to explore potential neuroanatomical differences between the resulting groups.

**Patients and methods:**

The study included episodic and chronic migraine patients seeking consultation at a specialized headache department. A neuropsychological evaluation protocol, encompassing validated standardized tests for cognition, anxiety, depression, perceived stress, and headache-related impact (HIT-6) and disability (MIDAS), was administered. Results from this evaluation were input into an automated K-means clustering algorithm, with a predefined K=2 for comparative purposes. A supplementary Voxel-based Morphometry (VBM) evaluation was conducted to investigate neuroanatomical contrasts between the two distinct grouping configurations.

**Results:**

The study involved 111 participants, with 49 having chronic migraine and 62 having episodic migraine. Seventy-four patients were assigned to cluster one, and 37 patients were assigned to cluster two. Cluster two exhibited significantly higher levels of depression, anxiety, and perceived stress, and performed worse in alternating and focalized attention tests. Differences in HIT-6 and MIDAS scores between episodic and chronic migraine patients did not reach statistical significance (HIT-6: 64.39 (±7,31) vs 62.92 (±11,61); *p*= 0. 42 / MIDAS: 73.63 (±68,61) vs 84.33 (±63,62); *p*=0.40). In contrast, patients in cluster two exhibited significantly higher HIT-6 (62.32 (±10,11) vs 66.57 (±7,21); *p*=0.03) and MIDAS (68.69 (±62,58) vs 97.68 (±70,31); *p*=0.03) scores than patients in cluster one. Furthermore, significant differences in grey matter volume between the two clusters were noted, particularly involving the precuneus, while differences between chronic and episodic migraine patients did not withstand correction for multiple comparisons.

**Conclusions:**

The classification of migraine patients based on neuropsychological characteristics demonstrates a more effective separation of groups in terms of disability compared to categorizing them based on the chronic or episodic diagnosis of ICHD-3. These findings could reveal biological changes that might explain differences in treatment responses among apparently similar patients.

**Supplementary Information:**

The online version contains supplementary material available at 10.1186/s10194-024-01734-1.

## Introduction

It is evident in the clinic that migraine affects some individuals more severely than others. To identify this subgroup of more severely affected patients, the International Classification of Headache Disorders (ICHD-3) defines a somewhat arbitrary boundary of 15 monthly headache days (8 of which must have migraine features) which was established by expert consensus [1]. Once this threshold is crossed, the diagnosis of chronic migraine is made. Chronic migraine has been traditionally considered the most burdensome form of migraine, as suggested by studies evaluating the quality of life and psychological health in the past [2, 3]. However, recent findings have shown that this pain-days threshold does not accurately capture the whole burden of illness, nor does it reflect the treatment needs of patients [4]. Therefore, a more precise separation system is warranted. Although redefining the headache days threshold could be a useful approach, the possibility arises that a more comprehensive phenotyping system involving non-painful variables might provide better outcomes. Indeed, there is a growing notion that migraine encompasses more than just headache, [5] highlighting the abovementioned possibility that clinical features other than pain could reveal the degree of underlying brain impairment more effectively than the number of days with headache alone.

Amongst the non-painful alterations tightly associated with migraine, cognitive and emotional and behavioral functioning disorders are of capital importance [5]. Cognitive disorders are observed in approximately 65% of migraine patients [6]. They usually consist of memory, attention, and information processing speed deficits, and are more commonly observed in severely affected patients [6, 7]. Anxiety and depression are also frequent in migraine and are associated with harder-to-treat cases [8, 9]. The association that exists between these psychiatric comorbidities and migraine is likely bidirectional, [10] and research has shown that a shared genetic background could be involved [11].

Considering this scenario, we questioned whether Cognitive and Psychological alterations would provide insightful information about brain alterations traducing into migraine-related disability. To do so, we evaluated the performance of a classification system based on these alterations in terms of disease severity, and how such classification aligns with ICHD-3 diagnosis. Additionally, we conducted a complementary voxel-based morphometry assessment to explore potential neuroanatomical differences between the resulting groups.

## Materials and methods

**Fig. 1:**
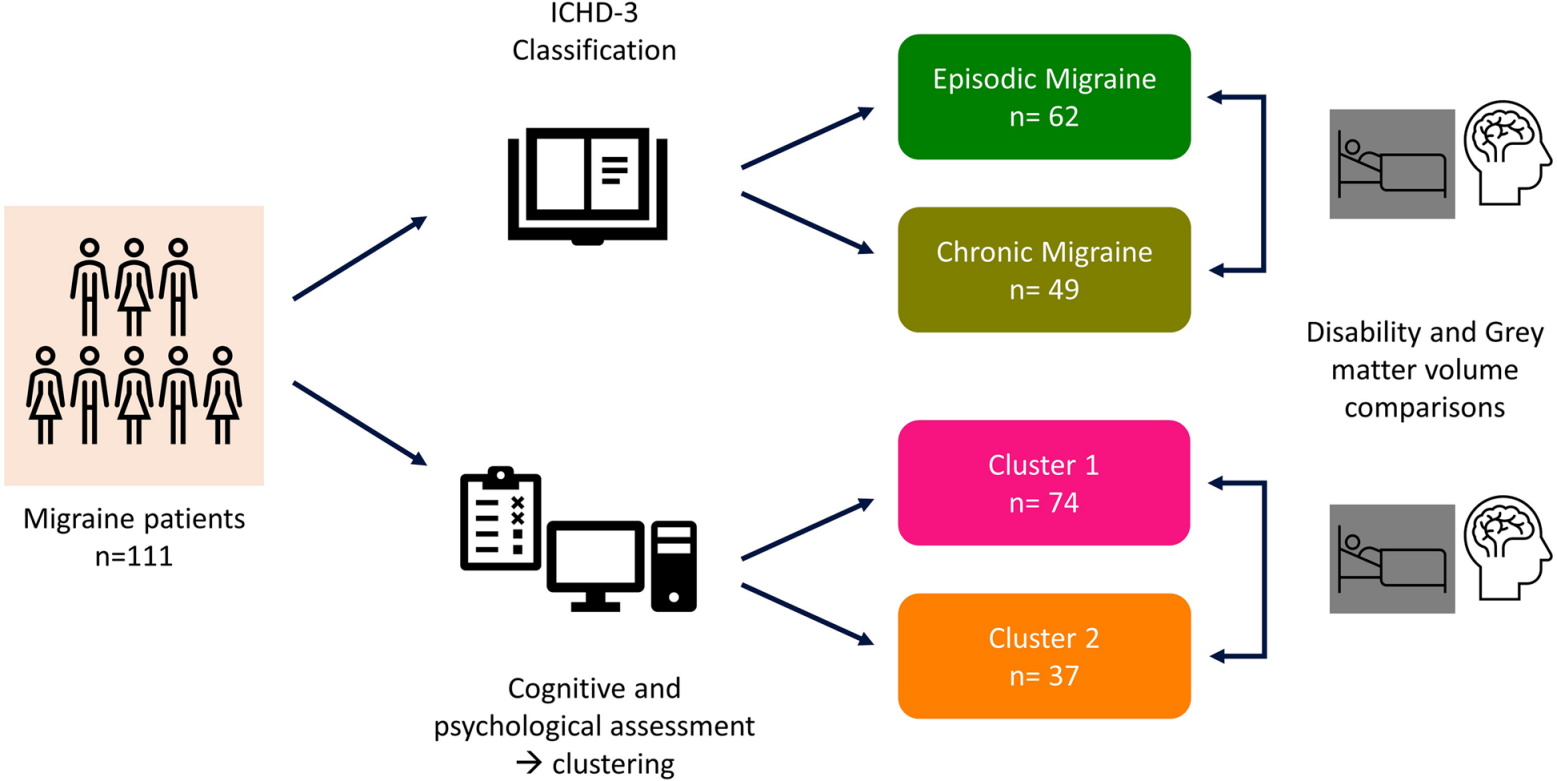
Graphical summary of the methodology used in this study

Figure 1 shows a graphical summary of the methodology used in this study.

### Patients

A convenience sample of patients consulting a specialized headache department from January 2019 to June 2022 was screened for inclusion. Only patients diagnosed with migraine in its chronic or episodic forms according to the ICDH-3, who satisfactorily completed a customized neuropsychological evaluation and a headache diary for at least one month were included. Patients who were pregnant, suffering from severe medical illnesses, or under treatment for psychiatric disorders were excluded.

Episodic or chronic migraine ICHD-3 diagnoses were performed by neurologists (PS & ML) based on information from paper diaries filled by patients. All included patients reported having a relatively stable headache frequency and intensity during the preceding months, similar to the one recorded in the headache diary. On these diaries, headache intensity, presence of nausea or vomiting, presence of photophobia and phonophobia, interference with daily activities, and intake of rescue medication were registered. Only the final diagnosis, and not the number or characteristics of monthly headache days and/or migraine days, were recorded in medical records.

### Neuropsychologic profile-based classification

The neuropsychological evaluation protocol comprised several tests. These tests were conducted to assess cognition, anxiety, depression, perceived stress, and headache-related impact and disability. The cognition tests involved the Verbal Paired Associates test from the Wechsler Memory Scale, the Wechsler Adult Intelligence Scale (WAIS) Reverse Digit Span subtest, the WAIS Digit-Symbol subtests, and the Trail Making Test A and B. Anxiety and depression were assessed through the Beck Anxiety Inventory (BAI) and Beck Depression Inventory-II (BDI-II) respectively. Perceived stress was evaluated using the Perceived Stress Scale 10-item version (PSS-10). Headache-related impact was assessed using the Headache Impact Test (HIT-6), and disability was measured using the Migraine Disability Assessment (MIDAS) (Table 1). Tests in the battery were selected based on the literature [7, 12–14] and the availability of locally adapted and validated versions, along with the existence of reliable reference data. Individual test results were converted into Z-scores based on normative data adjusted for age, gender, and education level. The Z-scores were then divided into three categories based on their range: mild alterations were between -1 and -1.5, moderate alterations were between -1.5 and -3, and severe alterations were below -3. Emotion and anxiety questionnaires have their own predetermined cut-off points. Evaluations were performed by a specialized psychologist (TCZ) in a dedicated room with adequate illumination, climatization, and sonorization. Patients were not using migraine medications known to impact cognitive abilities, nor had they utilized such medications in the preceding months. None of the patients were evaluated during a migraine attack.

**Table 1:** Psychological, cognitive, and severity aspects evaluated, and the tests employed

Psychological	
Depression	Beck depression inventory–II (BDI II)
Anxiety	Beck Anxiety Inventory (BAI)
Stress	Perceived Stress Scale (PSS- 10)
Cognitive	
Selective attention	Trail Making Test A
Alternating attention	Trail Making Test B
Focalized attention	Wechsler Adult Intelligence Scale (WAIS III) - Digit span
Verbal learning memory	Verbal Paired Associates test - Weschler Memory Scale
Delayed recall	Verbal Paired Associates test - Weschler Memory Scale
Processing speed	Wechsler Adult Intelligence Scale (WAIS III) – Digit symbol coding
Severity	
Headache impact	HIT-6
Migraine related disability	MIDAS

### Statistical analyses

Results from the neuropsychological evaluation protocol were introduced into an automated K-means clustering algorithm implemented in SPSS (IBM® SPSS® statistics V26). K-means clustering is a form of unsupervised machine learning feature-based grouping technique, which separated the sample into two differing clusters based on their characteristics. We *a priori* defined a K=2 which allowed us to run comparisons between the two classification systems.

Diagnostic proportions and differences in neuropsychological assessment battery results were compared using the chi-squared test (categories) and ANOVA (Z-scores). Results from HIT-6 and MIDAS scores were compared between the two ICHD-3 diagnosis groups (i.e., chronic versus episodic) and the two neuropsychological profile-based classification groups (i.e., cluster one versus cluster two) using unpaired t-tests. *p*-values below 0.05 were considered statistically significant.

### Voxel-based morphometry comparisons

Following the primary clinical part of this study, a complementary Voxel-based Morphometry (VBM) evaluation was performed to search for neuroanatomical contrasts between the two different grouping configurations (i.e., ICHD-3 diagnosis or automated K-means clustering).

High-resolution structural MRI images were obtained using a Philips Ingenia 3.0T scanner. A T1-weighted volumetric sequence with 1mm^3^ isotropic voxels was used for analysis. Forty patients (21 episodic and 19 chronic) from the original sample were originally included, but three had to be excluded after image visual inspection because of poor MRI quality (patient motion *n*=2, orthodontic appliances *n*=1). A final subset of 37 patients was available for evaluation. Following visual quality inspection, images were reoriented, co-registered with a template, segmented into different tissues (gray matter, white matter, cerebrospinal fluid, others), normalized, and smoothed (Gaussian Kernel, full-width half maximum (FWHM) of 8mm) as detailed in the software’s manual [15]. To enhance the normalization process and achieve a more precise alignment of the images, the Deformable Anatomical Registration Through Exponentiated Lie algebra (DARTEL) method was employed, minimizing inter-subject variability.

Image preprocessing and statistical tests were performed using SPM12 (http://www.fil.ion.ucl.ac.uk/spm) implemented in Matlab (The MathWorks 2021). Unpaired voxel-wise t-tests between groups derived from the ICHD-3 diagnosis or the neuropsychological profile-based K-means classification were carried out including age, sex, and total intracranial volume as covariates. A *p*-value <0.05 (Family-Wise Error (FWE) corrected) was considered statistically significant.

### Ethical considerations

The study was conducted following the principles of the Declaration of Helsinki and approved by the Ethics Committee of the School of Medicine University Hospital of the University of Córdoba. Written informed consent was not required for this study due to specific legal and ethical guidelines outlined in Law 25.326, Article 11(d) and Article 28, and Ministry of Health Resolution 1490/07, Chapter 4, Section 3. Therefore, all patients were provided comprehensive verbal explanations of the study and voluntarily consented to participate.

## Results

### Demographics and classifications

Neuropsychological evaluations of 111 patients were included for analysis. One-hundred-one patients (91%) were female and 10 (9%) were male. The mean age of participants was 38.5 ± 11.36 years (range:18-73).

In the whole sample, 49 patients were chronic, and 62 patients were episodic according to the ICHD-3. Amongst chronic migraine patients, 41 (84%) also fulfilled the criteria for medication overuse headache.

Regarding the neuropsychological-based classification, the automated clustering algorithm separated migraine patients into two different clusters: cluster one, with 74 patients, and cluster two, with 37 patients. The average silhouette Score was 0.36.

Forty-three out of the 74 patients in cluster one (58%) and 19 of the 37 patients (51%) in cluster two were diagnosed as suffering from episodic migraine, and the remaining were chronic. No significant differences in ICHD-3 diagnosis proportions were observed between the two clusters (Cluster 1: 58% episodic and 42% chronic; Cluster 2 51% episodic and 49% chronic. χ= 0.46; *p*=0.50).

Table 2 contains a brief overview of the descriptive features of the participants involved in the study.

**Table 2:** Descriptive characteristics of the study population separated based on the ICHD-3 (left columns) or K-means clustering (right columns)

	ICHD-3		Clustering	
	Episodic	Chronic	Cluster 1	Cluster 2
*n*=	62	49	74	37
Female	90.3%	91.8%	90.5%	91.9%
Male	9.7%	8.2%	9.5%	8.1%
Mean Age (SD)	37.9 (±11.1)	39.2 (±11.7)	38.27 (±10.9)	38.97 (±12.4)
Medication overuse		84%	34%	43%

### Neuropsychological variables

**Fig. 2:**
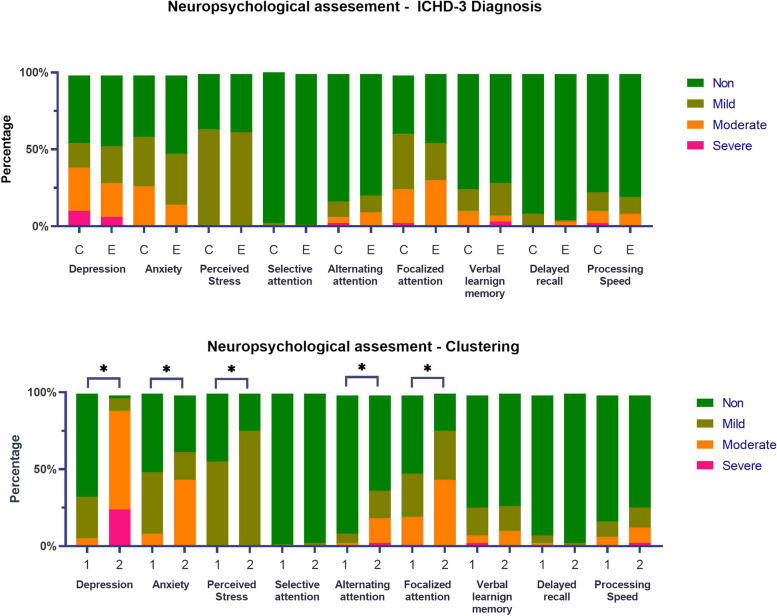
Psychological and cognitive features. Comparisons between ICHD-3 diagnoses (upper half) and neuropsychological-based clusters (lower half). The asterisk (*) denotes statistical significance

No significant differences were observed between chronic and episodic migraine patients in the neuropsychological variables evaluated. In contrast, cluster one and cluster two differed from each other in their levels of depression, anxiety, perceived stress, alternating attention, and focalized attention (all *p*<0.05). (Table 3 & Fig 2).

**Table 3:** Comparison of mean standardized values of the different cognitive and psychological domains evaluated between patients allocated to each cluster and patients classified according to the ICH-3. The asterisk (*) denotes statistical significance

	Cluster 1	SD	Cluster 2	SD	*p*-value	Episodic Migraine	SD	Chronic Migraine	SD	*p*-value
Depression	0.38	±0.59	2.11	±0.66	<0.01*	1.04	±1.08	0.89	±0.98	0.434
Anxiety	0.57	±0.64	1.05	±0.91	<0.01*	0.86	±0.82	0.63	±0.73	0.124
Perceived Stress	0.55	±0.50	0.76	±0.43	<0.05*	0.63	±0.49	0.61	±0.49	0.833
Selective attention	0.01	±0.12	0.03	±0.16	0.62	0.02	±0.14	0.02	±0.13	0.868
Alternating attention	0.12	±0.40	0.62	±0.95	<0.01*	0.27	±0.73	0.31	±0.64	0.753
Focalized attention	0.7	±0.82	1.19	±0.81	<0.01*	0.88	±0.83	0.85	±0.87	0.889
Verbal learning memory	0.38	±0.72	0.38	±0.68	1	0.35	±0.66	0.40	±0.73	0.677
Delayed recall	0.11	±0.39	0.03	±0.16	0.23	0.08	±0.28	0.08	±0.38	0.988
Processing Speed	0.24	±0.57	0.43	±0.80	0.15	0.35	±0.72	0.27	±0.61	0.565

### HIT-6 and MIDAS

**Fig. 3:**
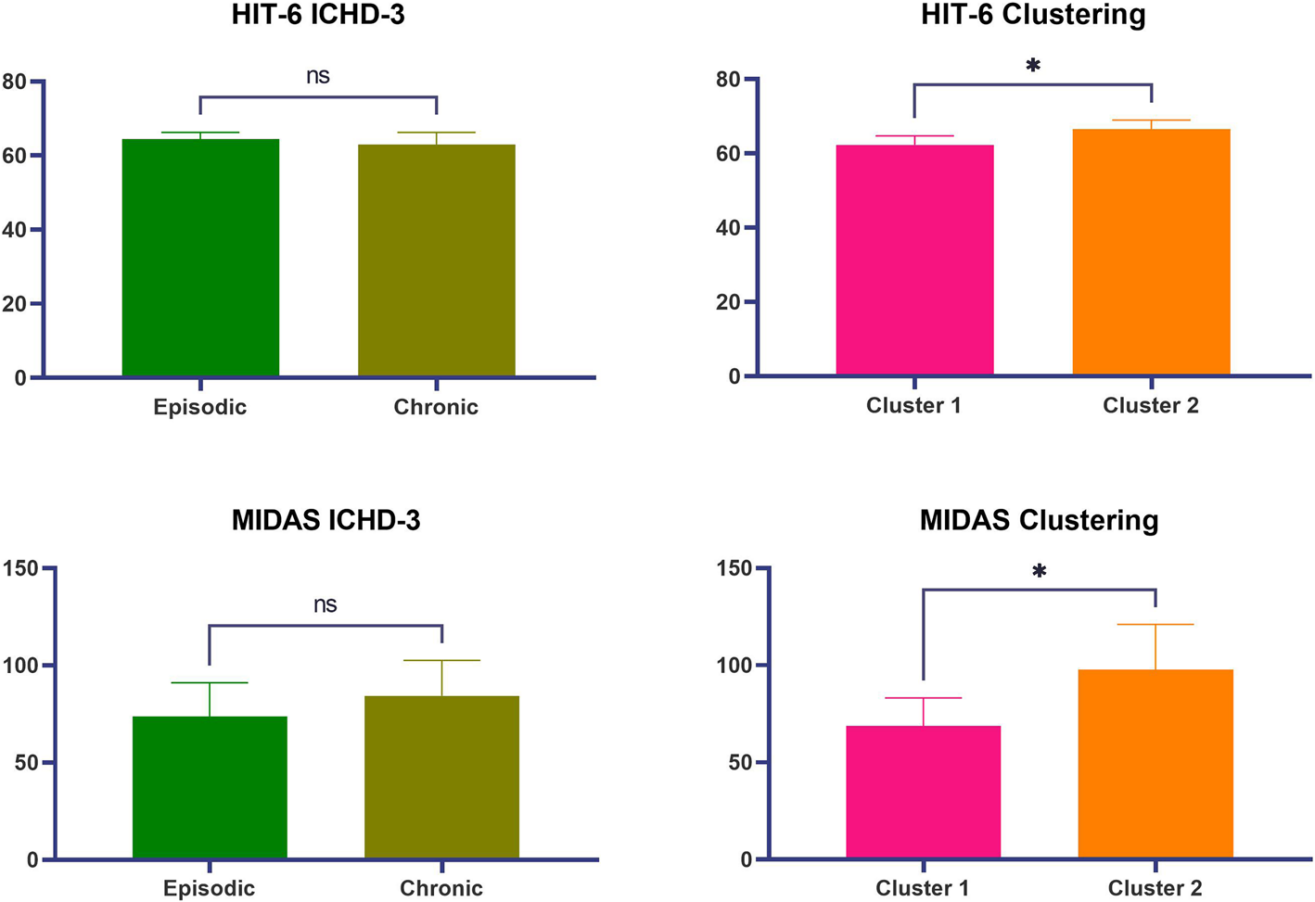
HIT-6 and MIDAS scores differences between groups according to the ICHD-3 (left) or the neuropsychological-based clusters (right). The asterisk (*) denotes statistical significance. ns= not significant

No significant differences were observed when comparing HIT-6 and MIDAS scores between episodic and chronic migraine patients (HIT-6: 64.39 (±7,31) vs 62.92 (±11,61); *p*= 0. 42 / MIDAS: 73.63 (±68,61) vs 84.33 (±63,62); *p*=0.40. Patients in cluster two had significantly higher HIT-6 (62.32 (±10,11) vs 66.57 (±7,21); *p*=0.03) and MIDAS (68.69 (±62,58) vs 97.68 (±70,31); *p*=0.03) scores than patients in cluster one (Fig 3).

### Voxel-based morphometry

**Fig. 4:**
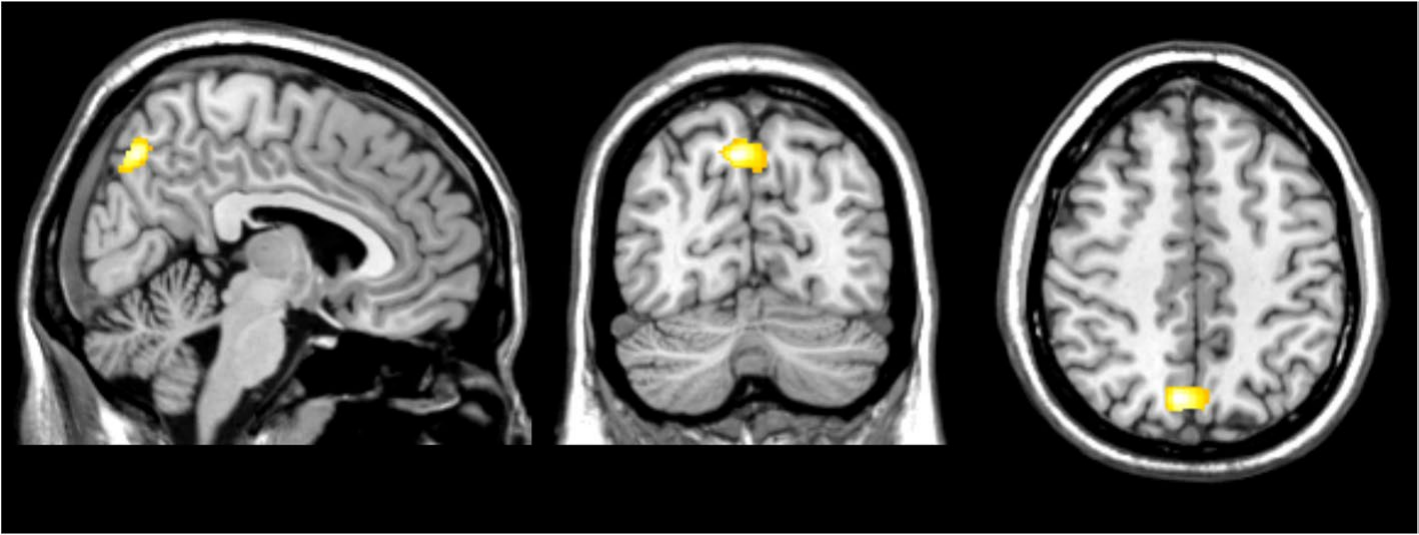
Regions of increased gray matter volume in Cluster One compared to Cluster Two overlaid onto the sagittal (x = 86), coronal (y = 56), and axial (z = 119) planes of a T1-weighted brain MRI template, radiologically oriented. The threshold has been set to *p*=0.001_unc_ for displaying purposes. Additional details can be found in the main text and Table 2

Voxel-based morphometry analyses controlled for multiple comparisons did not identify regions of significantly different grey matter volume between chronic and episodic migraine patients. Exploratory (*p*< 0.005 uncorrected) results for this comparison showed reduced gray matter in the right frontal cortex and left amygdala in chronic migraine patients. These are shown in the supplementary Figure (1). Conversely, VBM analysis evidenced significant differences in grey matter volume between cluster 1 and cluster 2 at a *p* < 0.05 FWE_corr_ statistical threshold, with cluster one exhibiting higher gray matter in the precuneus compared to cluster two (Table 4, Fig 4).

**Table 4:** Details of the region with increased gray matter volume in Cluster One (less disabled) with respect to Cluster Two

**Anatomy**	**Cluster size**	**T**	**Z equivalent**	**p(FWE-corr)**	**MNI coordinates**		
					**x (mm)**	**y (mm)**	**z (mm)**
Right Cerebrum, Parietal Lobe, Precuneus, Brodmann area 7	59	5,43	4,54	0,022	3	-72	46,5

## Discussion

In this study, we observed that separating migraine patients according to their neuropsychological characteristics results in more efficient segregation of groups in terms of disability than classifying them using the 15 monthly headache days threshold of the ICHD-3. The implications of these findings are discussed below Fig 4.

Both HIT-6 and MIDAS are validated measures of headache severity. Previous large-scale studies have described numerical differences between episodic and chronic migraine patients like those reported here. Yang et al. [16] described HIT-6 scores of 62.5 (± 7.8) and 60.2 (± 6.8) for chronic and episodic migraine patients respectively, a 2-point difference almost identical to the one we found in our study. Analogously, Bigal et al. [17] described an average MIDAS score of 34.9 in a group of patients with chronic migraine, and a mean of 19.3 in patients with episodic migraine, which represents a ~16 points difference, not far away from the ~11 points difference that we observed. Remarkably, in our study, differences between the two neuropsychological-driven clusters defined by an automated method were more pronounced and slightly more homogeneous than differences observed between chronic and episodic migraine patients. This led to statistical significance in the K-means classification system but not in the ICHD-3 comparison. Together, our results demonstrate that while distinctions in HIT-6 and MIDAS between episodic and chronic migraine patients are noticeable, the differences between the two neuropsychological-profile-based clusters we identified are steeper and more robust.

Our observations carry numerous potential implications for the clinical and research fields that merit further exploration. For instance, it would be intriguing to determine whether patients with a neuropsychological profile like that observed in cluster two of our study are less responsive to acute or prophylactic treatments, or if they benefit more from therapeutic approaches involving psychotherapy. Additionally, assessing whether this subset of patients incurs higher direct costs (such as medical visits, medications, and complementary examinations) or indirect costs (like absenteeism and presenteeism) related to the disease would be valuable. Notably, although performing a neuropsychological evaluation does not require important investments to be set up and should be feasible in all headache centers fulfilling the minimum recommended quality standards, [18] this practice is time and resource-consuming. However, our data indicate that it provides valuable information about patients, suggesting that it could potentially lead to cost-effective personalized care. This assumption should be tested in appropriately designed studies.

Concerning basic research, in the future, it would be valuable to examine whether patients with specific cognitive and psychological alterations resembling those of Cluster Two in our study exhibit differences in neurophysiological tests, neurotransmitter concentrations (i.e. serotonin, dopamine, or CGRP), or other neuroimaging modalities similar to the differences we observed. Recently, Tao et al. found that 83.33% of migraine patients with depression and none of the migraine patients without depression exhibit a hypoechogenic raphe [19]. Mickleborough and collaborators have extensively demonstrated the implication of attentional systems in migraine using electrophysiological techniques as well as functional magnetic resonance imaging [20, 21]. From a neurochemical perspective, a past study demonstrated that chronic migraine patients with comorbid depression exhibit reduced concentrations of gamma-aminobutyric acid (GABA) in the cerebrospinal fluid compared to their non-depressed counterparts, [21] and more recently, Alpuente et al. have described that salivary Calcitonin gene-related peptide (CGRP) concentrations in migraine patients are positively correlated with headache days, though the slope of this correlation becomes steeper in the presence of depression [22]. In other words, based on their data, a patient with migraine and comorbid depression who has 10 headache days a month has approximately the same CGRP levels as an undepressed migraine patient suffering from 28 monthly headache days [22]. Finally, it might be worth investigating whether a specific genetic background is associated with each of the two clusters, as indirect evidence points out [22]. In summary, converging research findings suggest that cognitive and psychological factors shape the migraine brain, and put together, might be the explanation behind our observations.

Of note, our voxel-based morphometry results when comparing episodic and chronic migraine patients aligned closely with those reported by Coppola et al. in 2017 [23]. Similar to their findings, we observed a reduction in grey matter volume in the left amygdala of chronic migraine patients, although, like in their study, it did not withstand correction for multiple comparisons. In contrast, grey matter volume differences between patients allocated to cluster one and patients allocated to cluster two using neuropsychological-based profiling resulted in significant differences in grey matter volume at a highly stringent statistical threshold. Contrasts involved a subregion of the precuneus characterized by its cognitive functions [24, 25] and by its tight connections with the temporoparietal junction, an area previously identified as a hub in attention processing that is highly relevant in migraine pathophysiology [21, 26, 27]. This could explain the involvement of this region in our analysis, although further research is warranted.

Our study has several limitations worth mentioning. Perhaps the major limitation was recording only migraine diagnosis and not the precise number of headache and migraine days, which hampered us from defining whether a particular cluster had more episodic migraine patients with high or low-frequency migraine. However, this limitation does not diminish the significance of our findings, as our study effectively contrasts with the broader current classification system, ICHD-3. Similarly, chronic migraine diagnosis requires a sustained elevated headache frequency for at least three months. However, in our clinic-based study, withholding treatment initiation for such a period to make a more precise diagnosis would have led to prolonged suffering for patients. Therefore, diagnoses were based on a one-month diary, usually covering the period between the appointment and the first visit or during the complementary examination period. Another potential limitation is our sample, which is limited to patients consulting a specialized headache service, impeding the extrapolation of our findings to the general population of patients. Finally, regrettably, there is a lack of reliable local reference data for most tests of executive logic functions. The currently available ones are too extensive to be implemented in everyday clinical practice. As a result, specific tests for executive logic functions were not included in the assessment. Therefore, although the TMT B and digit-symbol tests evaluate executive function to some extent, overcoming this limitation in the future would be of interest in the future.

## Conclusion

In summary, our study highlights the value of neuropsychological-based classification over the traditional chronic and episodic dichotomy in assessing disability in migraine patients. The robust distinctions observed between neuropsychological-driven clusters, surpassing differences between chronic and episodic migraine patients, underscore the clinical relevance of this approach. These findings could potentially lead to tailored treatments based on neuropsychological profiles, indicating a need for further exploration. Distinctive patterns were revealed through neuroanatomical investigations, with even greater differences when using the neuropsychological approach. This emphasizes cognitive nuances beyond conventional classifications. Despite study limitations, our results challenge existing paradigms and open avenues for future research into the structural, electrophysiological, biochemical, genetic, and therapeutic implications of neuropsychological profiles in migraines, advancing toward personalized treatment strategies.

### Supplementary Information

**Supplementary Material 1.** 

## Data Availability

Data related to this manuscript is not uploaded into any repository but will be made available upon reasonable request.
