# Supplementary figures and images for: Specific cognitive and psychological alterations are more strongly linked to increased migraine disability than chronic migraine diagnosis
Source: J Headache Pain. 2024 Mar 15;25(1):37. doi: 10.1186/s10194-024-01734-1 (PMC10941545; doi:10.1186/s10194-024-01734-1)

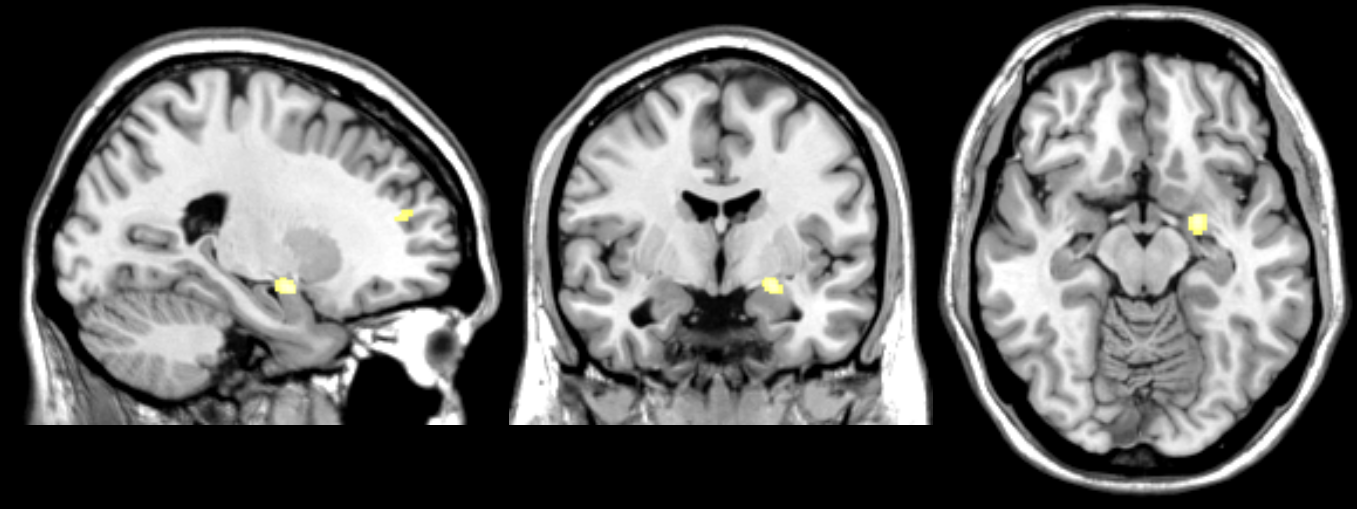

Supplement: Supplementary file 1 — Supplementary Material 1. [file 10194_2024_1734_MOESM1_ESM.png]
